# Effect of natural polyphenols in Chinese herbal medicine on obesity and diabetes: Interactions among gut microbiota, metabolism, and immunity

**DOI:** 10.3389/fnut.2022.962720

**Published:** 2022-10-28

**Authors:** Keyu Chen, Zezheng Gao, Qiyou Ding, Cheng Tang, Haiyu Zhang, Tiangang Zhai, Weinan Xie, Zishan Jin, Linhua Zhao, Wenke Liu

**Affiliations:** ^1^Department of Endocrinology, Guang’anmen Hospital, China Academy of Chinese Medical Sciences, Beijing, China; ^2^Institute of Metabolic Diseases, Guang’anmen Hospital, China Academy of Chinese Medical Sciences, Beijing, China; ^3^Graduate School, Beijing University of Chinese Medicine, Beijing, China; ^4^College of Traditional Chinese Medicine, Changchun University of Chinese Medicine, Changchun, China

**Keywords:** natural polyphenols, obesity, type 2 diabetes mellitus, gut microbiota, inflammation, traditional Chinese medicine

## Abstract

With global prevalence, metabolic diseases, represented by obesity and type 2 diabetes mellitus (T2DM), have a huge burden on human health and medical expenses. It is estimated that obese population has doubled in recent 40 years, and population with diabetes will increase 1.5 times in next 25 years, which has inspired the pursuit of economical and effective prevention and treatment methods. Natural polyphenols are emerging as a class of natural bioactive compounds with potential beneficial effects on the alleviation of obesity and T2DM. In this review, we investigated the network interaction mechanism of “gut microbial disturbance, metabolic disorder, and immune imbalance” in both obesity and T2DM and systemically summarized their multiple targets in the treatment of obesity and T2DM, including enrichment of the beneficial gut microbiota (genera *Bifidobacterium*, *Akkermansia*, and *Lactobacillus*) and upregulation of the levels of gut microbiota-derived metabolites [short-chain fatty acids (SCFAs)] and bile acids (BAs). Moreover, we explored their effect on host glucolipid metabolism, the AMPK pathway, and immune modulation *via* the inhibition of pro-inflammatory immune cells (M1-like Mϕs, Th1, and Th17 cells); proliferation, recruitment, differentiation, and function; and related cytokines (TNF-α, IL-1β, IL-6, IL-17, and MCP-1). We hope to provide evidence to promote the clinical application of natural polyphenols in the management of obesity and T2DM.

## Introduction

The global prevalence of metabolic diseases is rapidly increasing with the alteration of diet and lifestyle. The increasing consumption of high-calorie foods and displacement of leisure-time physical activities by sedentary activities lead to a positive energy balance (in which energy intake exceeds energy expenditure) and result in metabolic diseases. Obesity and type 2 diabetes mellitus (T2DM), two representative metabolic diseases, have substantial internal correlation in incidence trend and mechanism (1–3). Since 1980, the prevalence of obesity has doubled in 73 countries and increased in most other countries. In 2015, 604 million adults and 108 million children were diagnosed with obesity, according to a global survey of obesity in 195 countries. As an independent risk factor for T2DM, obesity-induced insulin resistance could also aggravate the occurrence of T2DM (4). According to the International Diabetes Federation Diabetes Atlas (10th edition), the global diabetes prevalence in 2021 was 537 million, and the number of patients with diabetes may rise to 783 million by 2045. The high incidence of obesity and T2DM, as well as their complications, has placed a huge burden on human health and medical expenses worldwide (5). Over the past few decades, obesity and T2DM have gradually come to the forefront of medical research. Thus, clinicians are attempting to explore their pathogenesis, as well as effective prevention and treatment methods. The pathogenesis of obesity and T2DM has been shown to be associated with interactions among gut microbial disturbance, metabolic disorders, and immune imbalance (6).

With the rapid development of high-throughput sequencing technology and emergence of gut microbial research, we have realized that gut microbiota play critical physiological roles in metabolism, especially energy extraction, and the control of local or systemic immunity. The disturbance of their composition and function appears to be involved in the pathogenesis of obesity and T2DM. Apart from the altered composition of gut microbiota, including decreased abundance of some beneficial microbiota, such as *Akkermansia muciniphila*, *Faecalibacterium prausnitzii*, *Bifidobacterium*, and *Blautia*, the main mechanisms of gut microbiota that contribute to the development of obesity and T2DM are based on their derived metabolites, including directly produced metabolites, such as short chain fatty acids (SCFAs), branched amino acids (BCAAs), aromatic amino acids (AAAs), and indirectly regulated metabolites, such as secondary bile acids (BAs) (7). These metabolites not only regulate glucose and lipid metabolism homeostasis but also maintain the balance of immunity as energy suppliers and signal molecules (8, 9). Therefore, the disturbance of gut microbiota composition and function aggravates the metabolic inflammatory state and accelerates the development of obesity and T2DM (10). As common core network mechanisms, targeting the interaction of “gut microbial disturbance, metabolic disorder, and immune imbalance” is of great importance to develop effective drugs for obesity and T2DM and clarify their mechanism and target of improving insulin resistance.

Recently, natural bioactive phytochemicals have been found to have potential health benefits for the prevention of obesity and T2DM. Polyphenols are a class of natural bioactive compounds derived from plants and have been shown to modulate physiological and molecular pathways involved in energy metabolism in obesity and T2DM (11). Therefore, natural polyphenols have gradually become a source of dietary supplements and new medicines owing to their potential in obesity and diabetes treatment. As an important source of polyphenols, Chinese herbal medicine (CHM) has been shown to have regulatory effects on metabolic disorders. The metabolic regulatory effects and intervention targets of various polyphenols contained in CHM need to be further revealed and verified.

In this review, we explain the network interaction mechanism of “gut microbial disturbance, metabolic disorders, and immune imbalance” in both obesity and T2DM. Focusing on this network mechanism, we studied the natural polyphenols derived from CHM and summarized their multiple targets for anti-obesity and anti-T2DM according to their efficacy. We hope to provide evidence to improve the clinical management of obesity and T2DM through the administration of natural polyphenols.

## Gut microbial disturbance, metabolic disorders, and immune imbalance: New insights into the core network mechanism of both obesity and type 2 diabetes mellitus

Metabolism and immunity were often considered the two main foundations for maintaining organismal homeostasis, wherein the function of metabolism is emphasized as the transformation of glucose, lipids, and energy, whereas immunity mainly protects against foreign invaders and removes endogenous hazardous substances (12, 13). The crosstalk between metabolism and immunity has been revealed in multiple diseases. Chronic low-grade inflammation is a hallmark of obesity and T2DM; metabolic disorders that often coexist with a systemic inflammatory state characterized by the upregulation of pro-inflammatory immune cells, including M1-like Mϕs, T helper (Th)1 cells, Th17 cells, CD8^+^ T cells, and antibody-producing B-2 cells, and downregulation of the quantity and proportion of anti-inflammatory immune cells, including M2-like Mϕs, Th2 cells, regulatory T cells (Tregs), IgM-producing B-1 cells, and several innate lymphocytes (ILCs) subsets (such as ILC2s and ILC3s). This is accompanied by increased levels of pro-inflammatory cytokines, such as tumor necrosis factor (TNF)-α, interleukin (IL)-1β, IL-2, IL-6, IL-17, and IFN-γ, in the circulation, adipose tissue, liver, and pancreas, which further disturb metabolism and aggravate pancreatic β-cell dysfunction and insulin resistance (14).

Many factors regulate the homeostasis of metabolism and immunity, such as gut microbiota according to their composition and associated metabolites (6). As the key factors that induce obesity and T2DM, high-fat diets (HFDs) can lead to a severe gut microbial disturbance, which is mainly manifested by the decrease in the abundance and proportion of beneficial bacteria, such as genera *Akkermansia*, *Faecalibacterium*, *Bifidobacterium*, and *Roseburia*, and increase in those of harmful bacteria of genera *Ruminococcus* and *Proteobacteria* (15). Moreover, HFD intake disrupts intestinal tight junction proteins and alters alkaline phosphatase activity, resulting in increased gut permeability and leaky gut syndrome. As a result, harmful bacteria or extracellular vesicles containing microbial genes can enter the host through the damaged intestinal barrier, often together with endotoxic substances, such as lipopolysaccharide (LPS). High levels of LPS can bind to the toll-like receptor (TLR) of macrophages and activate the downstream MyD88/JNK/IKK/NFκB pathway. Then, the macrophages exhibit pro-inflammatory M1 polarization and release several pro-inflammatory cytokines, such as TNF-α, IL-1β, IL-6, and inducible nitric oxide synthase (iNOS) (16), thereby causing a chronic low-grade inflammation of glucolipid metabolic tissues and organs, along with insulin resistance or insulin secretion dysfunction in islet β-cells (17, 18).

In addition to direct translocation and stimulation, gut microbiota-associated metabolites participate in “immunometabolism” regulation. The disturbance of gut microbial composition could accelerate its associated metabolite disorders, which are involved in the microbial modification of dietary component-derived metabolites, such as SCFAs, BCAAs, and AAAs, directly and microbial modification of host-derived metabolites, such as secondary BAs, indirectly. These metabolites are also involved in the crosstalk between host metabolism and immunity. Through this ordered interwoven mechanism network, host homeostasis is cooperatively maintained; otherwise, it will lead to the development of many diseases, including obesity and T2DM (7).

Short-chain fatty acids, including acetate, propionate, and butyrate, are a class of fatty acids with the composition of 1–6 carbon atom backbones. They are the derivatives of gut microbiota formed by the intestinal microbial fermentation and degradation of dietary fibers and polysaccharides. SCFAs are involved in maintaining intestinal mucosal integrity, controlling glucose, lipid, and energy metabolism, and regulating the immune system and inflammatory responses based on multiple pathways, such as the G protein-coupled receptor family, and epigenetic modification by acting as a histone deacetylase inhibitor (19). Most acetates and propionates are mainly derived from the gut microbiota of phylum Bacteroidetes, such as species *Blautia hydrogenotrophica*, *Methanobrevibacter smithii*, *Eubacterium hallii*, and *Eubacterium cylindroides*, whereas phylum Firmicutes, the main producers of butyrate, includes species *Faecalibacterium prausnitzii*, *Roseburia intestinalis*, and *Ruminococcus gnavus* (20). The abundance of SCFA-producing bacteria and downregulated levels of SCFAs in both dysmetabolic mice and humans with obesity and T2DM have been extensively reported (21, 22).

The production and modification of BCAAs and AAAs are regulated by gut microbiota. Among BCAAs, valine, isoleucine, and leucine are essential amino acids derived from the diet through gut microbial conversation. *Prevotella copri* and *B. vulgatus* are their main producers (23). The exact role of BCAAs in metabolism in obesity and T2DM is a double-edged sword. On the one hand, they could control the thermogenesis of brown adipose tissues *via* SLC25A44 transporters in the mitochondria and boost energy metabolic status (24). On the other hand, BCAAs show a positive association with visceral fat accumulation, which may aggravate obesity and T2DM (25). As representative AAAs, tryptophan and indole-derivative metabolites also participate in the regulation of metabolism and immunity. Tryptophan can be transformed into indole, indole acetic acid, indole-3-lactic acid (ILA), and indole-3-propionic acid based on the catalytic effect of tryptophan decarboxylase (TDC), tryptophanase, and indole-lactic acid dehydrogenase derived from genera *Clostridium*, *Bifidobacterium*, *Lactobacillus*, and *Peptostreptococcus* (20, 26, 27). As classic aromatic hydrocarbon receptor (AhR) ligands, tryptophan and its indole-associated derivatives maintain the integrity of the intestinal barrier by accelerating the proliferation and renewal of intestinal epithelial cells to consolidate the intestinal physical barrier, limiting the access of gut microbes and LPS (28, 29). Moreover, several intestinal immune cells, such as dendritic cells, ILC3s, Th17 cells, and intraepithelial lymphocyte γδ T cells (γδ T cells), that are all high-expression of AhR and sensitively regulated by tryptophan and its indole-associated derivatives. The tryptophan-AhR axis can alleviate inflammation through the production and secretion of cytokines IL-10 and IL-22 (30, 31). Obesity and T2DM have a similar phenomenon, which is the reduced capacity of the microbiota to metabolize tryptophan into AhR agonists and impaired gut barrier to accelerate systemic chronic inflammation (32).

In addition to the above dietary component-derived metabolites, gut microbiota modify host-derived metabolites and mediate the production of BAs. Serum cholesterol is converted into primary BAs, such as chenodeoxycholic acid and cholic acid in hepatocytes, through neutral (classic) and alternative (acidic) pathways, mediated by key enzymes, such as CYP7A1, CYP7B1, CYP8B1, and CYP27A1, and secreted in the gastrointestinal tract. Thus, it could regulate the levels of serum cholesterol based on the production of primary BAs (33). In the gastrointestinal tract, primary BAs can be further transformed into secondary BAs by conjugation to glycine or taurine through bile salt hydrolase (BSH) derived from genera *Bacteroides*, *Lactobacillus*, *Bifidobacterium*, *Clostridium* (clusters XIVa and XI), and *Eubacterium* (34, 35). Then, secondary BAs can be absorbed through the intestine and regulate glucolipid metabolism by binding to farnesoid X receptor (FXR) and Takeda G protein-receptor-5 (35). BAs generally can: (1) promote glycogen synthesis and insulin sensitivity in the liver; (2) increase insulin secretion by the pancreas; (3) facilitate energy expenditure, especially in the liver, brown adipose tissue, and muscles; and (4) mediate satiety in the brain (36). Therefore, BA metabolism not only results in the production of primary BAs but also regulates downstream production of secondary BAs and plays a crucial role in maintaining metabolic homeostasis to ameliorate obesity and T2DM. Thus, they are a potential key regulatory target for alleviating metabolic diseases.

Based on the network interaction mechanism of “gut microbial disturbance, metabolic disorders, and immune imbalance” of obesity and T2DM, multi-target intervention can play a synergistic role in comprehensively alleviating obesity and T2DM. Among them, natural polyphenols, whether from dietary sources or traditional herbal sources, have been reported to improve obesity and diabetes *via* the regulation of these three core mechanisms.

## Natural polyphenols derived from Chinese herbal medicine: The key active compounds that alleviate obesity and type 2 diabetes mellitus

### Molecular structure and pharmacodynamic material basis of natural polyphenols

Polyphenols are a large family of secondary metabolites derived from plants, mainly synthesized through shikimic and malonic acids. Polyphenols can effectively suppress immune responses to ameliorate hyperglycemia and hyperlipidemia (37) based on their potential anti-inflammatory and antioxidant effects, as well as modulating the core gut microbiome (38).

The multiple functions of polyphenols are attributed to their unique basement of chemical structure and molecular activity. Polyphenols are defined by benzene rings and several phenolic hydroxyl groups and commonly classified into two main groups: flavonoid and non-flavonoid polyphenols. Most flavonoids have a common parent nuclear structure: diphenylpropane skeleton (C6-C3-C6). It is characterized by an oxygenated heterocycle (C-ring) generated by two aromatic rings (A-ring and B-ring) with 3 C atoms (39). According to different attachment sites of the B-ring, oxidation degree of the three-carbon chain, and whether the three-carbon chain forms a ring, flavonoids are mainly divided into flavonoids, flavonols, isoflavones, chalcones, flavanones, and anthocyanidins (40). Non-flavonoids are mainly composed of phenolic acid, stilbene, lignan, and coumarin. Polyphenols can be different in structure and biological activity owing to the number of phenolic rings, the structure of the linkages between phenolic rings, or the differences in the substitution groups linked to the rings (41). They display a variety of properties, including the formation of covalent or non-covalent compounds conjugated with other phenols, amines, lipids, and sugars, as well as the formation of a stable pentacyclic chelate through complexation reaction with metal ions. These physicochemical properties are responsible for their strong antioxidant and free radical scavenging ability. Hence, they play a crucial role in inhibiting the growth, proliferation, and metastasis of tumors, as well as progression of neurodegenerative and cardiovascular disorders, diabetes, and COVID-19 (42).

Polyphenols widely vary in molecular weight, from simple compounds of small molecular weight, such as phenolic acids, to complex polymers of large molecular weight, such as procyanidins, ranging from 500 to 5000 Da (43). More than 8000 polyphenols, with different characteristics, have been identified in nature so far (44). They are widely present in vegetables, fruits, legumes, cereals, herbs, and products derived from plants such as coffee, tea, red wine, oils, and chocolate (41). This indicates that the Mediterranean diet, including vegetables, fruits, and cereals that contain abundant dietary polyphenols, is beneficial to health by ameliorating various chronic diseases, such as obesity and T2DM.

### Polyphenols alleviate obesity and type 2 diabetes mellitus according to clinical and experimental evidence

Based on limited human studies and animal experiments, a diet rich in polyphenols can reduce fasting plasma glucose (FPG) levels and postprandial hyperglycemia, as well as improve acute insulin secretion and insulin sensitivity (45). A meta-analysis showed that many polyphenols derived from diets have non-linear associations in dose-response studies, which suggested that diary polyphenols are associated with a reduced risk of T2DM (46). Medicinal plants are essential sources of natural polyphenols and have been identified to exhibit anti-diabetic effects and lower blood lipid levels in patients suffering from diabetes. A randomized, double-blind, placebo-controlled trial (RCT) that included 240 subjects with criteria of prediabetes showed that none developed T2DM after 9 months of curcumin treatment, whereas 16.4% of the subjects in the placebo group were diagnosed with T2DM. Further evaluation revealed that the anti-diabetic effect of curcumin was based on the protection of islet β cells (47). Another RCT study, which involved 70 Japanese subjects with overweight or obesity, concluded that 12 weeks of daily administration of 9 g of onion powder rich in quercetin resulted in a substantial decrease in visceral fat area by 5.1 cm^2^ compared with that of the placebo group (48). Daily ingestion of bread enriched with 0.05% of a 1:1 mixture of quercetin and epicatechin for more than 3 months, substantially alleviated glucolipid metabolic disorders owing to a mean reduction of total cholesterol of 14.2 mg/dL, low-density lipoprotein cholesterol of 21.4 mg/dL, total triglycerides of 50.18 mg/dL, and FPG of 119.44 mg/dL (49). More evidence from another meta-analysis of 37 RCTs also showed that administration of anthocyanins for more than 8 weeks at doses higher than 300 mg/day (312.6 mg/day, median: 320 mg/day) remarkably reduced FPG levels, postprandial plasma glucose level, glycated hemoglobin level, and homeostatic model assessment of insulin resistance compared with those of the subjects who administered anthocyanins for ≤8 weeks at 193.9 mg/day (median: 160 mg/day) [Fallah et al. (50)]. Besides, the anthocyanins also showed a significant effect in reduction of BMI and T2DM risk (51, 52). Through combing the anti-obese and anti-diabetic clinical evidence of above 3 representative natural polyphenols such as curcumin, quercetin and anthocyanin, we also found the effects of natural polyphenols on immune regulation and antioxidant stress, including inhibiting the levels of hs-CRP, IL-6 and MDA, etc. That indicated their potential anti-metainflammatory mechanisms and encouraging researchers to further explore about it (Table 1).

**TABLE 1:** Clinical evidence (RCT and meta-analysis) of 3 representative natural polyphenols (curcumin, quercetin, and anthocyanin) in alleviating obesity and T2DM.

Intervention	Study design	Patients	Sample size (intervention/ control)	Dose	Control	Intervention duration	Outcomes	References
Curcumin	Randomized, double-blind, placebo-controlled trial	T2DM	44 (21/23)	1500 mg/d	placebo	10w	***(endpoint of curcumin vs. baseline of curcumin, changes, p)*** **TG↓**: 109 ± 36 vs. 124 ± 36, −14.2 ± 30.6 mg/dl, *p* = 0.03; **hs-CRP↓**: 2.9 ± 2.9 vs. 4.4 ± 5.8, −2.5 ± 4.3 mg/l, *p* = 0.002; **serum adiponectin↑**: 64 ± 3 vs. 53 ± 8, 12.1 ± 7.7 ng/ml, *p* = 0.0001; **weight and FPG↓**: NA.	(153)
Curcumin	Randomized, double-blind, placebo-controlled trial	pre-diabetes/T2DM	33 (15/18)	180 mg/d	placebo	6m	***(endpoint of curcumin vs. baseline of curcumin, changes, p)*** **OX—LDL↓**: It was increased in the placebo group (1.4 [1.1, 1.7] vs. 1.3 [1.0, 1.4], *p* = 0.0024), but not increased in the curcumin group (1.1 [1.0, 1.3] vs. 1.1 [1.0, 1.3], *p* = 0.722).	(154)
Curcumin and/or omega-3 PUFA	2 × 2 factorial, randomized, double-blinded, placebo-controlled study	pre-diabetes/BMI: 25–45 kg/m^2^	64 (curcumin:15/ Placebo:16/LCn-3PUFA:17/double active: 16)	180 mg/d	placebo	12w	***(changes of curcumin vs. changes of placebo, p)*** **FPI↓**: NA, *p* = 0.05; **TG↓**: NA, *p* = 0.019; **AIP↓**: NA, *p* = 0.025.	(155)
Curcumin	Randomized, double-blind, placebo-controlled trial	pre-diabetes/BMI: 25–45 kg/m^2^	29 (14/15)	180 mg/d	placebo	12w	***(changes of curcumin vs. changes of placebo, p)*** **FPI**↓: −1.9 ± 0.6 vs.0.1 ± 0.4 μIU/L, *p* = 0.0115; **HOMA-IR↓**: −0.3 ± 0.1 vs. 0.01 ± 0.05, *p* = 0.0142; **IAPP↓**: −2.0 ± 0.7 vs. 0.4 ± 0.6 ng/mL, *p* = 0.0163; **GSK-3β↓**: −2.4 ± 0.4 vs. −0.3 ± 0.6 ng/mL, *p* = 0.0068.	(156)
Curcumin	Randomized, double-blind, placebo-controlled trial	overweight and obese female adolescents (aged 13–18 years)	60 (30/30)	500 mg/d	placebo	10w	***(endpoint of curcumin vs. baseline of curcumin, p)*** **BMI↓**: 31.43 ± 2.84 vs. 31.00 ± 2.85 kg/m2, *p* = 0.019; **WC↓**: 100.31 ± 1.14 vs. 97.86 ± 1.13 cm, *p* = 0.008; **HC↓**: 114.18 ± 2.77 vs. 113.17 ± 2.77 cm, *p* = 0.030; **HDL↓**: 48.93 ± 1.17 vs. 50.77 ± 1.18 mg/dL, *p* = 0.042; **TG/HDL↓**: 2.49 ± 1.16 vs. 2.19 ± 0.95, *p* = 0.021.	(157)
Curcumin	Randomized, double-blind, placebo-controlled trial	overweight and obese female adolescents	60 (30/30)	500 mg/d	placebo	10w	***(changes of curcumin vs. changes of placebo, p)*** **IL-6↓**: −15.28 ± 41.92 vs. 6.05 ± 22.42 ng/L, *p* = 0.018; **TAC↑**: 0.01 ± 0.03 vs. −0.01 ± 0.02 mmol/L, *p* = 0.001; **MDA↓**: −69.58 ± 88.01 vs. −18.17 ± 55.77 μmol/L, *p* = 0.009.	(115)
Nano curcumin	Randomized, double-blind, placebo-controlled trial	T2DM with diabetic sensorimotor polyneuropathy	80 (40/40)	80 mg/d	placebo	8w	***(changes of curcumin vs. changes of placebo, p)*** **HbA1c↓**: −0.70 ± 0.88 vs. 0.03 ± 0.57%, *p* < 0.001; **FPG↓**: −14.80 ± 27.73 vs. 4.80 ± 31.27 mg/dL, *p* = 0.004; **total score of neuropathy↓**: −2.07 ± 2.1 vs. −0.6 ± 1.5, *p* < 0.001; **total reflex score↓**: −0.65 ± 1.6 vs. 0, *p* = 0.04;	(158)
Curcumin mixture	Randomized, double-blind, placebo-controlled trial	BMI: 25–45 kg/m^2^/FPG: 100–125 mg/dL	80 (40/40)	800 mg/d (200 mg curcumin, 120 mg phosphatidylserine, 480 mg phosphatidylcholine and 8 mg piperine)	placebo	8w	***(endpoint of curcumin vs. baseline of curcumin, p)*** **BMI↓**: 26.3 ± 1.4 vs. 27.1 ± 1.8 kg/m^2^, *p* ≤ 0.05; **WC↓**: 89 ± 4 vs. 94 ± 7 cm, *p* ≤ 0.05; **FPG↓**: 101 ± 6 vs. 108 ± 9 mg/dL, *p* ≤ 0.05; **FPI↓**: 15 ± 3 vs. 18 ± 5, *p* ≤ 0.05; **HOMA-IR↓**: 3.8 ± 1.1 vs. 4.9 ± 1.1, *p* ≤ 0.05; **TG↓**: 151 ± 16 vs. 185 ± 21 mg/dL, *p* ≤ 0.05; **HDL↑**: 44 ± 4 vs. 40 ± 3 mg/dL, *p* ≤ 0.05; **LAP↓**: 62 ± 10 vs. 64 ± 14, *p* ≤ 0.05; **HSI↓**: 35 ± 4 vs. 38 ± 5, *p* ≤ 0.05; **FLI↓**: 54 ± 9 vs. 57 ± 11, *p* ≤ 0.05; ***(endpoint of curcumin vs. endpoint of placebo, p)*** **FPI↓**: 15 ± 3 vs. 18 ± 5, *p* ≤ 0.05; **HOMA-IR↓**: 3.8 ± 1.1 vs. 4.7 ± 1.4, *p* ≤ 0.05; **TG↓**: 151 ± 16 vs. 157 ± 19 mg/dL, *p* ≤ 0.05; **FLI↓**: 54 ± 9 vs. 57 ± 10, *p* ≤ 0.05;	(159)
Curcumin	Randomized, double-blind, placebo-controlled trial	overweight/obese women (BMI: 25–35 kg/m^2^) with polycystic ovary syndrome	60 (30/30)	500 mg/d	placebo	6w	***(endpoint of curcumin vs. endpoint of placebo, changes, p)*** **FPI↓**: 12.35 ± 6.79 vs. 15.42 ± 8.09, −3.06 ± 6.44 μU/mL, *p* = 0.020; **QUICKI↓**: 0.33 ± 0.03 vs. 0.32 ± 0.02, 0.01 ± 0.01, *p* = 0.003; **HOMA-IR↓**: 3.26 ± 2.26 vs. 3.95 ± 2.30, −0.69 ± 1.87, *p* = 0.067;	(160)
Curcumin	Meta-analysis	NA	876 (53% women)				**BW↓**: Weighed Mean Difference (WMD): −1.14 kg, 95% CI: −2.16, −0.12, *p* = 0.02; **BMI↓**: WMD: −0.48 kg/m^2^, 95% CI: −0.78, −0.17, *p* = 0.002.	(161)
	Meta-analysis	PCOS	168				**FPG↓**: MD: −2.77, 95% CI: −4.16 to −1.38; *p* < 0.001; *I*^2^ = 0%; **FPI↓**: MD: −1.33, 95% CI: −2.18 to −0.49; *p* = 0.002; *I*^2^ = 0%; **HOMA-IR↓**: MD: −0.32, 95% CI:-−0.52 to −0.12; *p* = 0.002; *I*^2^ = 0%; **QUICKI↑**: MD: 0.010, 95% CI: 0.003–0.018; *p* = 0.005; *I*^2^ = 69%; **HDL↑**: MD: 1.92, 95% CI: 0.33–3.51; *p* = 0.018; *I*^2^ = 0%; **CHO↓**: MD: −12.45, 95% CI: −22.05 to −2.85; *p* = 0.011; I2 = 32%.	(162)
Quercetin and epicatechin	randomized, double-blind, placebo-controlled study	MS:at least 3 of the following risk factors TG > 160 mg/dL; FPG > 100 mg/dL; HDL < 45 mg/dL; LDL > 130 mg/dL; abdominal obesity (WC > 102 cm for men or >88 cm for women); BMI >29	156 (78/78)	993 μmol/d	placebo	3m	***(endpoint of bread with flavonoid mixture (BF) vs. control bread (CN), p)*** **CHO↓**: 206.2 ± 15.7 vs. 220.4 ± 15.4 (mg/dl), *p* < 0.05; total cholesterol **LDL↓**:123.5 ± 21.1 vs.144.9 ± 23.4 (mg/dl), *p* < 0.05; **CHO↓**:164.73 ± 19.25 vs. 214.91 ± 20.1 (mg/dl), *p* < 0.05; **FPG↓**: 101.23 ± 13.25 vs. 120.67 ± 10.9 (mg/dl), *p* < 0.05;	(49)
Quercetin aglycone	randomized, double-blind, placebo-controlled, parallel-group study	BMI:23–30 kg/m^2^	54 (27/27)	60 mg/d	placebo	12w	***(endpoint of Quercetin-rich onion vs. changes of placebo, p)* VFA whose lower HDL-C** ↓: −5.8 ± 5.8 vs. −5.8 ± 5.8 (cm^2^), *p* = 0.046; visceral fat area.	(48)
Anthocyanins	randomized, double-blind, placebo-controlled, crossover intervention study	healthy individuals (BMI: 21–29.9 kg/m^2^) with a high fat meal	24	320.4 ± 0.7/d	placebo	5 h post-HFM	***(changes of 5 h AUC post-HFM of anthocyanins vs. 5 h AUC post-HFM of placebo, p)*** **Postprandial endotoxemia↓:** **LPS AUC↓**: 0.38 ± 0.15 vs. 0.68 ± 0 EU/mL for 5 h, *p* < 0.05; **LBP AUC↓**: 7.9 ± 2.0 vs.16.4 ± 3.0 μg/mL for 5 h, *p* < 0.03; **postprandial glucolipid metabolism↓:** **PPG AUC↓:** 23.0 ± 4.4 vs. 39.6 ± 8.0 mg/dL for 5 h, *p* < 0.02; **CHO AUC↓**: 31.2 ± 7.0 vs. 57.0 ± 10.0 mg/dL for 5 h, *p* < 0.06 **TG AUC↓**: 161.5 ± 18.5 vs. 203.5 ± 29.6 mg/dL for 5 h, *p* = 0.03 **Inflammation and oxidative stress biomarkers in PBMC↓:** Increases in IL-8 (51%), TNFα (45%), and NOX4 (74%, *p* < 0.04) mRNA levels in placebo group whereas no significant HFM-mediated increases in TNFα and NOX4 were observed in anthocyanins group.	(163)
Anthocyanins and berry fruits	Meta-analysis	T2DM	/	/	/	/	Dietary anthocyanin consumption was associated with a **15% reduction of T2DM risk** (summary RR = 0.85; 95% confidence interval (CI): 0.80–0.91; *I*^2^ = 14.5%); Consumption of berries was associated with an **18% reduction of T2DM risk** (summary RR = 0.82, 95% CI: 0.76–0.89; *I*^2^ = 48.6%); The risk of T2DM was decreased by 5%, with a 7.5 mg/day increment of dietary anthocyanin intake (RR = 0.95; 95% CI: 0.93–0.98; *I*^2^ = 0.00%) or with a 17 g/day increment of berry intake (RR = 0.95, 95% CI: 0.91–0.99; *I*^2^ = 0.00%), respectively.	(51)
Anthocyanins	Meta-analysis	obesity	/	/	/	/	**BMI↓**: **−**0.36 kg/m^2^, 95% CI = **−**0.58–0.13; *I*^2^ = 0%; *p* = 0.002	(52)
Anthocyanins	Meta-analysis	T2DM	/	/	/	/	**FPG↓**: **−**2.70 mg/dl, 95% CI: **−**4.70 to **−**1.31; *p* < 0.001; **2-h PG↓**: **−**11.1 mg/dl, 95% CI: **−**18.7 to **−**3.48; *p* = 0.004; **HOMA-IR↓**: **−**0.54, 95% CI: **−**0.94 to **−**0.14; *p* = 0.008; R**esistin↓**: **−**1.23 μg/l, 95% CI: **−**2.40 to **−**0.05; *p* = 0.041; **PAI-1↓**: **−**5.09 μg/l, 95% CI: **−**9.45 to **−**0.73; *p* = 0.022	(50)

↑ Means up-regulation after natural polyphenols intervention; ↓ means down-regulation after natural polyphenols intervention; NA, non-reported. TG, triglyceride; hs-CRP, high-sensitivity C-reactive protein; FPG, fasting plasma glucose; OX, oxidative; LDL, low density lipoprotein; PUFA, polyunsaturated fatty acid; LCn, long chain number; HDL, high density lipoprotein; FPI, fasting plasma insulin; AIP, atherogenic index of plasma; HOMA-IR, homeostasis model assessment of insulin resistance; IAPP, islet amyloid polypeptide; GSK-3β, glycogen synthase kinase-3 β; BMI, body mass index; WC, waist circumference; HC, hip circumference; TAC, total antioxidant capacity; MDA, malondialdehyde; HbA1c, glycosylated hemoglobin A1c; HOMA-IR, homeostatic model assessment for insulin resistance; LAP, lipid accumulation product; HSI, hepatic steatosis index; FLI, fatty liver index; BW, body weight; PCOS, polycystic ovary syndrome; MS, metabolic syndrome; QUICKI, quantitative insulin sensitivity check index; LPS, lipopolysaccharide; LBP, lipopolysaccharide binding protein; PPG, postprandial plasma glucose; 2-h PG, 2-h postprandial glucose; PAI-1, plasminogen activator inhibitor-1.

Additionally, experimental evidence revealed that polyphenols ameliorated metabolic disorders. Multiple doses of puerarin, such as 140, 150, and 200 mg/kg/day, remarkably decreased body weight, normalized glucose level, improved glucose tolerance, and protected β-cells in animal experiments (53, 54). Moreover, emodin ameliorated hyperglycemia and dyslipidemia owing to the alleviation of insulin resistance and improvement of insulin sensitivity in a concentration- and time-dependent manner (19). Furthermore, the ingestion of salvianolic acid (A or B) decreased insulin intolerance and downregulated the levels of FPG, triglycerides, as well as free fatty acids levels in mice/rats (55–60). In summary, natural polyphenols derived from CHM effectively alleviate metabolic disorders, which further supports their clinical application.

## Multiple targets of natural polyphenols in metabolic disorders based on regulation of gut microbiota, metabolism, and immunity

In the prevention and treatment of metabolic disorders, such as obesity and T2DM, the exact efficacy of natural polyphenols has been widely confirmed in several RCTs and animal experiments. However, their regulatory mechanism has not been systematically summarized. A growing body of literature has revealed that the mechanisms of polyphenols involve many aspects, such as regulation of gut microbiota, metabolism, and immunity, including anti-inflammatory and antioxidant effects. Herein, we explore the network interaction mechanism of “gut microbial disturbance, metabolic disorders, and immune imbalance” in both obesity and T2DM, taking several representative natural polyphenols, such as curcumin, quercetin, puerarin, emodin, and salvianolic acid derived from CHM as examples, in an attempt to systematically explain the mechanism of action of natural polyphenols on body weight and glucose levels and to clarify the targets of polyphenols for supporting industrial transformation in the next step (Table 2).

**TABLE 2:** Multiple targets of gut microbiota, metabolism, and immunity of natural polyphenols derived from CHM for the management of obesity and T2DM.

		Curcumin	Quercetin	Puerarin	Emodin	Salvianolic acid	Common targets	References
Gut Microbiota	Beneficial Bacteria(↑)	Genera: *Bifidobacteria, Lactobacilli*	Genera: *Bifidobacterium, Bacteroides, Clostridia, Lactobacillus, Coprococcus_1, Anaerovorax, Ruminiclostridium_9, Mucispirillum, Roseburia, Tyzzerella*	Genera: *Lactobacillus, Barnesiella, Clostridium IV, Prevotella, Akkermansia muciniphila*	Genera: *Lactobacillus spp., Akkermansia, Verrucomicrobia, Erysipelotrichia*	/	Genera: *Bifidobacterium, Akkermansia, Lactobacillus*	(59, 66–70, 72–75, 90)
	Harmful Bacteria(↓)	Phylum: Firmicutes/Bacteroidetes ratio*;* Genera: *Prevotellaceae, Coriobacterales, Enterobacteria, Rikenellaceae, Actinobacteria, Proteobacteria*	Genera: *E. coli, Proteobacteria, Enterococcus, Fusobacterium*	/	/	Genera: *Proteobacteria, Deferribacteres*	Genera: *Proteobacteria*	
Gut Microbial Metabolites	SCFAs(↑)	Acetate, propionate and butyrate in feces and serum (↑)			/	/	acetate, propionate, butyrate	(75, 85, 86)
	Bile Acids(↑)	Expression of CYP7A1 and the production of primary BAs (↑)				/	CYP7A1	(71, 87–89)
Glycolipid Metabolism	Liver	AMPK, SREBP1c, ChREBP, FAS, CPT1, ACAT (↑)	AMPK (↑)	AMPK (↑)	AMPK (↑)	/	AMPK	(95–99)
	Adipose Tissue	AMPK and Wnt/β-catenin signaling pathway (↑)	AMPK (↑); Fnta, Pon1, Pparg, A1dh1b1, Apoa4, Abcg5, Gpam, Acaca, Cd36, Fdft1, Fasn	/	AMPK and CPT 1 (↑); SREPB 1 and FAS (↓)	AMPK-Sirtuin 1 signaling pathway (↑); UCP-1 (↑); lncRNA-Hsd11b1, lncRNA-Vmp1		(96, 100–105)
	Skeletal Muscle	AMPK-GLUT4 pathway (↑)		/	/	/		(106, 107)
	Islet β cells	Akt (↑); Nuclear translocation of Foxo1 and ER stress (↓)	Apoptosis and ferroptosis *via* Sirt3 (↓)	GLP-1R (↑)	/	Amyloid formation (↓)		(54, 108–111)
Immunity Regulation	Mϕs (M1-like Mϕs,↓) (M2-like Mϕs,↑)	NF-κB (p65), Stat1, Tlr4, Il6 (↓)	AMPK (↑); ERK1/2, JNK, p38MAPK, TLR4/NF-κB, tnfα, il6, il1β, cox2 (↓)	NF-κB, CCL2, CCL4, CCL5, CXCR4 (↓)	TREM2 (↑); TLR4/MyD88/NF-κB, NFκB/IRF5/STAT1, IRF4/STAT6 (↓)	MAPKs/NF-κB pathways: JNK, ERK 1/2, IκB, and NF-κB p65 (↓)	NF-κB	(117–126)
	NLRP3 inflammasome (↓)	AMPK (↑); TLR4/MyD88/NF-κB (↓);	/	/	/	TXNIP/ NLRP3;TXNIP/ ChREBP; NF-κB	NF-κB	(128–132)
	T lymphocytes (Th1 and Th17,↓) (Th2 and Treg,↑)	Potential effects on rebalance of Th1/Th2 and Th17/Treg balance and further exploration in the treatment of obesity and T2DM				/	/	(132, 134–140)
	Cytokines (pro-inflammation,↓) (anti-inflammation,↑)	TNF-α, IL-6, IL-1β, MCP-1, INF-γ, IL-17 (↓); IL-4, IL-10, IL-22 (↑)					/	Derived from the references of Mϕs and T lymphocytes
	Oxidative Stress(↓)	ROS (↓); Nrf2-Keap1 pathway (↑)	ROS (↓);GSH, SOD and GPx (↑)	MDA (↓); SOD, GPx (↑)	MDA (↓);SOD (↑); Nrf2, AMPK (↑)	ROS, MDA (↓)/SOD (↑)	ROS, MDA, SOD	(92, 130, 142–146)

↑ Means up-regulation after natural polyphenols intervention; ↓ means down-regulation after natural polyphenols intervention; / means NA, non-reported.

### Interactions between polyphenols and gut microbiota to alleviate obesity and type 2 diabetes mellitus

A complex and dynamic interplay between polyphenols and gut microbiota contributes to the overall homeostasis of the intestinal environment. First, the bio-transformation of polyphenols into active metabolites (phenolics) was promoted by gut microbiota (61). A variety of bacterial species, such as *Bifidobacterium catenulatum*, *Lactobacillus sp.*, *Escherichia coli*, *Bacteroides sp.*, *Eubacterium sp.*, *Enterococcus caccae*, and *Ruminococcus gauvreauii* participated in catalyzing phenolics metabolism (62, 63). Taking curcumin as an example, its metabolic bio-transformation was related to reduction, methylation, demethoxylation, hydroxylation, and acetylation, and its related products, such as tetrahydrocurcumin, propanoic acid, and dihydroferulic acid, were mainly regulated by the genera *Blautia*, *Bifidobacterium*, *Lactobacillus*, and *Faecalibacterium* (64, 65). Similarly, polyphenol intake affects gut microbial composition and function to some extent. By enriching beneficial bacteria, reducing the abundance of harmful bacteria, and decreasing the levels of their related metabolites, they further play a role in regulating glycolipid metabolism.

Curcumin remarkably shifted the ratio between beneficial and harmful bacteria, by upregulating the abundance of genera *Bifidobacteria*, *Lactobacilli*, and other butyrate-producing bacteria and decreasing the abundance of genera *Prevotellaceae*, *Coriobacterales*, *Enterobacteria*, and *Rikenellaceae*, which are often associated with the onset of chronic diseases (66–68). Tetrahydrocurcumin effectively increased the expression of pancreatic glucagon-like peptide-1 (GLP-1) to promote insulin secretion and reduce FPG levels. Its hypoglycemic effect was attributed to reducing the relative abundance of genera *Actinobacteria* and *Proteobacteria* and phylum *Firmicutes/Bacteroidetes* ratio (69).

Quercetin administration altered the composition of gut microbiota through downregulation of the abundance of harmful genera, such as *E. coli*, *Proteobacteria, Enterococcus*, and *Fusobacterium* and upregulation of the abundance of beneficial genera, such as *Coprococcus*, *Ruminiclostridium*, *Roseburia*, *Bifidobacterium*, *Bacteroides*, *Clostridia*, and *Lactobacillus* (70, 71). Similarly, puerarin and emodin, natural polyphenols, regulated gut microbiota by increasing the abundance of genera *Lactobacillus* and *Akkermansia*. Furthermore, puerarin upregulated the levels of beneficial gut microbiota, such as *Barnesiella*, *Clostridium IV*, and *Prevotella*. Emodin also substantially increased the abundance of some intestinal barrier-protecting bacteria, including *Verrucomicrobia* and *Erysipelotrichia* (72–75). In addition to the gut microbial alteration with salvianolic acid anti-obesity intervention, it could inhibit the expression of *Proteobacteria* and *Deferribacteres* (59).

**FIGURE 1:**
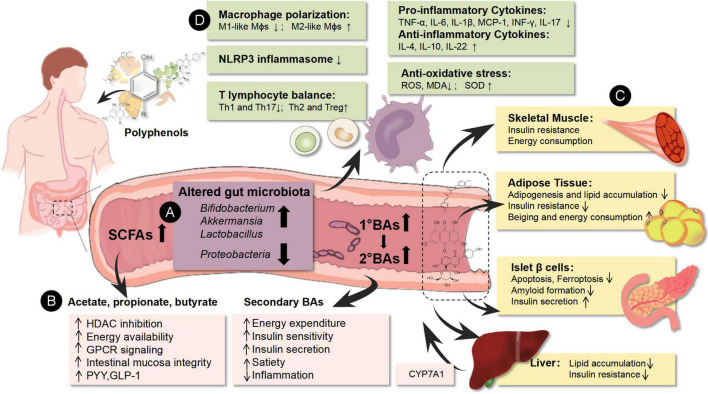
Environmental factors could affect the epigenetics of immune cells to generate tissue inflammatory states and induce metabolic disorders. The pathogenesis of obesity and T2DM is associated with gut microbial disturbance, metabolic disorder, and immune imbalance. Based on network mechanisms, natural polyphenols showed multiple targets in the treatment of obesity and T2DM, including enrichment of beneficial gut microbiota (*Bifidobacterium, Akkermansia*, and *Lactobacillus*) and decrease of the abundance of harmful gut microbiota (*Proteobacteria*), increase of gut microbiota-derived metabolites (SCFAs, BAs), as well as regulation of the host glucolipid metabolism of pancreatic islets, adipose tissue, liver, and skeletal muscle. Meanwhile, the mechanisms of natural polyphenols are also related to immunity rebalance through polarization of Mϕs and the ratio of T lymphocyte subtypes. They could down-regulate the pro-inflammatory immune cells (M1-like Mϕs, Th1, and Th17 cells) and their related cytokines (TNF-α, IL-6, IL-1β, MCP-1, INF-γ, and IL-17) as well as up-regulate the anti-inflammatory immune cells (M2-like Mϕs, Th2, and Treg cells) and their related cytokines (IL-4, IL-10, and IL-22). Besides, their antioxidant effects contributed to immunoregulation. ↑ means up-regulation after natural polyphenols intervention; ↓ means the down-regulation after natural polyphenols intervention. **(A)** Represents the purple area; **(B)** represents the pink area; **(C)** represents the yellow area; **(D)** represents the green area.

Natural polyphenols could substantially increase the abundance of beneficial gut microbiota and decrease the expression of some pathogenic bacteria. Polyphenols contributed to the enrichment of *Bifidobacterium*, *Akkermansia*, and *Lactobacillus*, as well as inhibition of *Proteobacteria*. These microbiota play a crucial in maintaining intestinal homeostasis and relieving metabolic disorders. *Bifidobacterium*, the widely used probiotic, has been proven to be safe and effective in lowering the levels of FPG and total cholesterol, with an inverse association with metabolic disorders, as well as low-grade inflammation, insulin resistance, and T2DM in mice and humans (76, 77). The same effect was observed with *Akkermansia*, which is considered a next-generation probiotic. The key mechanism of both microbiota is that they are producers of SCFAs, and the effects of SCFAs have been summarized above (78). Furthermore, *Akkermansia* participated in the repair of the intestinal barrier through upregulating the expression of ZO-1 and occludin *in vivo* and *in vitro* (74). As a pivotal source of acetic acid production, *Lactobacillus* was also regarded as a probiotic, and its curative effect was detected in obese subjects with T2DM through an RCT of fecal microbiota transplantation (77). On the contrary, Proteobacteria are the most consistently reported obesity-associated phylum, and they upregulate the risk for the development of metabolic diseases, including atherosclerosis, insulin resistance, and T2DM, as potential drivers of low-grade inflammation (79) (Table 2 and Figure 1A).

Not limited to the 4-core gut microbiota described above, other gut microbial changes were detected after polyphenols intervention, such as *Clostridium, Prevotellaceae, Enterobacteria, Roseburia*, and *Rikenellaceae*. They were also reported to have an association with obesity and T2DM (80). Therefore, the influence on gut microbial composition and function is, at least partially responsible, for the effect of polyphenols on metabolic disorders.

### Two perspectives on metabolic regulation by polyphenols: Gut microbiota-derived metabolites and host metabolism

The regulatory effect of polyphenols on metabolism can be mainly divided into two levels. On the one hand, they indirectly regulate the level of metabolites derived from gut microbiota, which will further affect the metabolic state. The regulation of the structure of gut microbiota has been systematically summarized above. On the other hand, polyphenols and their derivatives directly regulate the target organs of glucose and lipid metabolism, such as pancreatic islets, adipose tissue, liver, and skeletal muscle, thereby improving obesity and T2DM. This multi-faceted metabolic regulation determines the wide application of polyphenols in the prevention and treatment of various metabolic diseases (Table 2 and Figures 1B,C).

The representative receptors of SCFAs, such as G-protein-coupled receptors 41 (GPR41)/free fatty acid receptor 3 (FFAR3), GPR43/FFAR2, and GPR109a, are mainly expressed on gut enteroendocrine cells, adipocytes, and several immune cells (81). SCFAs could stimulate the secretion of gut hormone peptide YY and GLP-1 to inhibit appetite and increase insulin secretion and sensitivity through binding to GPR41 and GPR43 (82, 83). In addition, butyrate could repair the gut barrier as an energy supplier and participate in the anti-inflammatory immunity homeostasis, such as mediating M2-like macrophages and decreasing the levels of pro-inflammatory cytokines (TNF-α, IL-1β, and IL-6) (84). Natural polyphenols, including curcumin, quercetin, and puerarin, could upregulate the levels of SCFAs in feces and serum, based on their regulatory effects on gut microbial composition, thereby alleviating metabolic disorders (75, 85, 86).

Natural polyphenols also participate in BAs metabolism through multiple pathways. First, curcumin, quercetin, puerarin, and emodin could upregulate the expression of CYP7A1 and accelerate the production of primary BAs to alleviate obesity by reducing cholesterol levels (71, 87–89). In the gut, although the role of polyphenols in the synthesis of secondary BAs has not been reported, curcumin, quercetin, and puerarin have been reported to enrich the abundance of genera *Bacteroides*, *Bifidobacterium*, and *Lactobacillus*; these are important sources of BSH, which mediates the production of secondary BAs (65, 75, 90). Therefore, polyphenols may be indirectly involved in the synthesis of secondary BAs based on gut microbiota. Moreover, curcumin could also upregulate the expression of receptors, such as FXR, to enhance the role of BAs in regulating downstream glucose and lipid metabolism (91, 92).

Not limited to indirect influence through gut microbial metabolites (SCFAs and BAs), the glucolipid metabolic regulation of polyphenols also includes direct activation of the liver, fat, skeletal muscle, and pancreas. AMP-activated protein kinase (AMPK), as a highly conserved and classic metabolic stress sensing protein kinase, plays a crucial role in maintaining glucolipid metabolism and energy homeostasis of multiple organs, especially the liver, fat, and skeletal muscles (93, 94). It is also the target of polyphenols in alleviating obesity and T2DM. The activation of AMPK by curcumin, quercetin, puerarin, and emodin could inhibit liver lipid accumulation and insulin resistance (95–98). In addition, the key hepatic lipid-regulating enzymes, including sterol regulatory element-binding protein 1c, carbohydrate response element-binding protein, fatty acid synthase, carnitine palmitoyltransferase 1, and acyl-CoA:cholesterol acyltransferase, were also positive triggered in regulating hepatic lipid metabolism by curcumin (99). In adipose tissue, curcumin could inhibit adipogenesis and fat mass based on the activation of AMPK and Wnt/β-catenin signaling and downstream targets, such as c-Myc and cyclin D1 (100, 101). In addition to the upregulation of AMPK, quercetin could modify the expression of genes related to lipid metabolism, including *FNTA*, *PON1*, *PPARG*, *APOA4*, *ABCG5*, *GPAM*, *ACACA*, *CD36*, *FDFT1*, and *FASN* (96, 102). For the HFD-induced obesity mouse model, emodin alleviated lipid accumulation in white adipose tissue through AMPK activation and its downstream targeting enzyme, such as up-regulation of CPT1, and downregulation of SREBP1 and FAS (103). Browning of white adipose is crucial for metabolism based on reducing insulin resistance (IR) and increasing energy consumption. Salvianolic acid could promote adipocyte thermogenesis and energy expenditure *via* the activation of the AMPK-Sirtuin-1 pathway and upregulation of Uncoupling protein-1 (UCP-1) (104). Moreover, salvianolic acid B may play an anti-obesity role by adjusting the expression of mRNAs correlated with inflammatory response and energy metabolism of brown adipose tissue through regulating lncRNA-Hsd11b1 and lncRNA-Vmp1 (105). Similarly, the AMPK/GLUT4 pathway is important for glucose transport and uptake in skeletal muscles, and curcumin and quercetin could regulate glucose metabolism in skeletal muscles through this pathway (106, 107).

As the main source of insulin, maintaining the normal function of islet β-cells is also one of the mechanisms of polyphenols in the treatment of obesity and T2DM. The beneficial actions of curcumin on islet β-cells are related to the suppression of apoptosis, improvement of glucose-induced insulin secretory function by Akt activation, inhibition of nuclear translocation of Foxo1, and alleviation of ER stress (108). Quercetin could inhibit oxidative stress-induced apoptosis *via* Sirt3 regulation, as well as ferroptosis of pancreatic islet β-cells (109, 110). The protective mechanisms of puerarin and salvianolic acid are attributed to the activation of GLP-1R signaling and inhibition of the amyloid formation of human islet amyloid polypeptide, respectively (54, 111).

### Anti-inflammatory mechanism of polyphenols contributes to their effects on metabolic disorders

The role of chronic low-grade inflammation in the pathological process of insulin resistance and metabolic disorders has been widely explored. Inflammatory responses can not only directly promote the occurrence and development of diseases, but also serve as a mediator for the regulation of diseases by the alteration of gut microbiota and organism metabolism. Therefore, the gut microbiota and their derived metabolites could directly affect the intestinal mucosal immunity, and the excessive accumulation of some metabolites such as FAs could also accelerate oxidative stress and inflammation in metabolic tissues and organs (16). Targeted immune regulation and inhibition of chronic inflammation are the key ways to improve metabolic disorders, and polyphenols have multi-targeted effects on relieving inflammation, including inhibiting the proliferation and differentiation of pro-inflammatory immune cells, as well as the production and secretion of pro-inflammatory cytokines, and alleviating up-stream oxidative stress (Table 2 and Figure 1D).

The inhibitory effect of natural polyphenols on the overall pro-inflammatory profile of immune cells is beneficial to the treatment of metabolic disorders. In addition to regulating the quantity and proportion of immune cells, polyphenols alleviate the pro-inflammatory phenotype by regulating some key inflammation-related pathways and the secretion of cytokines and chemokines. Polyphenols, such as curcumin and quercetin, substantially decreased the serum levels of pro-inflammatory cytokines, including TNF-α, IL-6, IL-1β, and MCP-1 in the treatment of obesity and T2DM (112–115). These cytokines were mainly derived from M1-like Mϕs, which play a core role in metabolic inflammation. As an important component of innate immunity, the Mϕs could polarize into two functional phenotypes including pro-inflammatory M1-like Mϕs and anti-inflammatory M2-like Mϕs. The balance of M1-like Mϕs and M2-like Mϕs could remarkably influence the inflammatory state and insulin resistance of adipose tissue, liver, and pancreatic islets (116). Curcumin could substantially inhibit the M1-like Mϕs infiltration in white adipose tissue (WAT), as well as reduce the expression of key pro-inflammatory genes, such as NF-êB p65 subunit (p65), STAT1, TLR4, and IL-6 (117). These mechanisms were substantially associated with the abundance of some beneficial gut microbiota, such as genera *Lactococcus*. The effect of regulating the polarization of Mϕs is also expressed with other polyphenols interventions, including quercetin, puerarin, and emodin during the treatment of obesity or T2DM. These polyphenols could bind to TLRs and regulate downstream multiple inflammatory signaling pathways such as NF-kB, activated protein-1 (AP-1), mitogen-activated protein kinases (MAPKs), c-Jun N-terminal kinase (JNK), Janus kinase/Signal transducer and activator of transcription (JAK/STAT), and other signaling pathways. Therefore, the inhibition of M1-like Mϕs and upregulation of M2-like Mϕs could decrease the levels of pro-inflammatory TNF-α, IL-6, IL-1β, and MCP-1 in the serum, adipose tissue, liver, and pancreatic islets, as well as alleviating IR and maintaining insulin secretion (118–126). The NOD-like receptor pyrin domain-containing 3 (NLRP3) inflammasome is also a key mediator of Mϕs involvement in the inflammatory process in obesity and T2DM (127). Curcumin could directly restrain the assembly of the NLRP3 inflammasome or inhibit the activation of the NLRP3 inflammasome through several classic pathways such as TLR4/MyD88/NF-kB and AMPK (128, 129). Quercetin and salvianolic acid A also inhibited the NLRP3 inflammasome, and the mechanism of salvianolic acid A was mainly based on the TXNIP/NLRP3 and TXNIP/ChREBP pathways (130, 131). In addition, salvianolic acid A ameliorated early stage atherosclerosis development of T2DM by inhibiting the activation of NLRP3 inflammasome and NF-kB pathways in the aortic tissues of Zucker Diabetic Fatty rats (132).

In addition to the polarization of Mϕs, the balance of the T lymphocyte subtype is also crucial to immunity homeostasis. An imbalanced profile of T lymphocytes was demonstrated in the pathological process of obesity and T2DM, through the upregulation of pro-inflammatory Th1 and Th17 cells, as well as the downregulation of anti-inflammatory Th2 and Treg cells. Consequently, T lymphocyte-derived cytokines were altered, as evidenced by the increase in the levels of TNF-α, INF- γ, and IL-17, as well as the decrease in IL-4, IL-10, and IL-22. Although the regulatory role of polyphenols in T lymphocytes in the context of obesity and T2DM has not been fully revealed, the effect of polyphenols on T lymphocytes has been widely reported in several other chronic inflammatory diseases, which further suggests that T lymphocytes are one of the immune mechanisms to alleviate obesity and T2DM by polyphenols (132). Curcumin inhibited Th17 differentiation and upregulated Treg to rebalance the ratio of Th17/Treg in type 2 diabetic mice with colitis (133). Moreover, the effects of curcumin on Th1/Th2 and Th17/Treg balance in other autoimmune diseases, such as psoriasis, inflammatory bowel disease, asthma, and allergy were also reported (134). Quercetin regulates Th17/Treg balance owing to multiple mechanisms, which include AHR, Tim-3, MAPK-TLR4, TLR4-MyD88-NF-kB, and PPAR-γ signaling pathways (135–138). Puerarin and emodin have also shown potential effects on Th1/Th2 and Th17/Treg balance in disorders other than obesity and T2DM (139, 140).

It is worth noting that oxidative stress is closely related to inflammatory processes, especially in obesity and T2DM. The accumulation of reactive oxygen species (ROS), which enhances inflammation, is aggravated by metabolic disorders. As natural antioxidants, polyphenols exhibited antioxidant activity owing to their ROS-scavenger ability and inhibition of ROS-generating enzymes, such as NOX and iNOS. They could also upregulate superoxide dismutase (SOD), glutathione (GSH), and glutathione peroxidase (GPx) (141). Curcumin reduced ROS production owing to its effect on nicotinamide adenine dinucleotide phosphate oxidase, increasing the activity of antioxidant enzymes, and modulation of the Nrf2-Keap1 pathway (142, 143). Additionally, quercetin restored the levels of GSH, SOD, and GPx in the serum and liver during the treatment of non-alcoholic fatty liver disease and T2DM (92, 144). The antioxidant effects of puerarin, emodin, and salvianolic acid A were attributed to inhibition of MDA and upregulation of SOD (130, 145, 146).

### Crucial common interactive mechanisms of polyphenols against metabolic disorders

Targeting the network interaction of “gut microbial disturbance, metabolic disorder, and immune imbalance,” we could summarize that the natural polyphenols have some common mechanisms in the management of obesity and T2DM, including enrichment of beneficial gut microbiota (genera *Bifidobacterium*, *Akkermansia*, and *Lactobacillus*), upregulation of the levels of gut microbiota-derived metabolites (SCFAs, BAs), and modulation of the host glucolipid metabolism by directly targeting the AMPK pathway, as well as rebalancing the immunity through inhibition of pro-inflammatory immune cells (M1-like Mϕs, Th1, and Th17 cells) and their related cytokines (TNF-α, IL-1β, IL-6, IL-17, and MCP-1) (Table 2 and Figure 1). Curcumin substantially alleviated dietary obesity and upregulated energy expenditure. These multiple mechanisms involved increasing relative abundance of the *Lactococcus*, *Parasutterella*, and *Turicibacter* genera. Moreover, the altered microbial composition accelerated the metabolism of curcumin into curcumin-O-glucuronide. Curcumin supplementation further reduced total macrophage infiltration and inflammation in WAT, consistent with reduced mRNA levels of M1 (Cd80, Cd38, and Cd11c) and the expression of other key pro-inflammatory genes, such as NF-kB p65, STAT1, TLR4, and IL-6, in WAT (117). Remarkably, despite research providing a glimpse of the common interactive mechanisms of polyphenols against metabolic disorders, larger-scale and higher-quality RCT studies, as well as precise and comprehensive network mechanism exploration, are still needed for further exploration.

## Discussion and perspectives

In general, with the global pandemic of metabolic diseases, especially obesity and T2DM, the huge pressure on prevention and treatment, and the burden of medical expenditure are also driving the research and development of drugs for the management of these diseases. In this review, we focused on several natural polyphenols derived from CHM and systematically combed their effect and mechanisms on obesity and T2DM. On the basis of summarizing the clinical evidence that confirmed their effect on alleviating metabolic disorders, crucial common interactive mechanisms and intervention targets, which involved gut microbiota, metabolism, and immunity, were revealed and supported the clinical application of polyphenols (Figure 1).

Considering the great potential of polyphenols in the fields of nutraceuticals and medications, it is important to explore more productive, environmental, and wholesome extraction methods. Extraction techniques of percolation, decoction, heat reflux extraction, Soxhlet extraction, and maceration are conventional. Although these methods have the advantages of convenience, they also pose negative effects like high time consumption, huge energy consumption, and low extraction rate (147). Non-conventional extraction methods include ultrasound extraction, microwave extraction, supercritical extraction, enzyme-assisted extraction, pulsed electric field, and high-voltage electric discharge, which are distinctive in targeting different polyphenols. Further, the separation method represented by membrane separation, is advanced and environmentally friendly, as the direction of precision and accuracy in the future (148). As active components of CHM, polyphenols are easily destroyed by oxidation under heat through traditional decoction processes such as salvianolic acid (*Salvia miltiorrhiza*), gallic acid (*Hypophylla*), and quercetin (*Ramulus mori*). Compared with traditional herb extraction methods, microwave-assisted extraction (MAE) and ultrasound-assisted extraction (UAE) show their strengths. Found in the Cassia extraction of fistula by Castro-López C, MAE has a higher extraction efficiency than maceration, decoction, and UAE (149). Sungpud C found that, compared with mangosteen peel, UAE can not only reduce the extraction time but also enhance the bioactivities of the extract (150).

In addition to the extraction methods, a limitation to extensive clinical application is the low bioavailability. Bioavailability is usually defined as the fraction of an ingested nutrient or compound that reaches the systemic circulation and the specific sites where it can exert its biological activity. The intervention effects of natural polyphenols in humans depend on their absorption, distribution, metabolism, and elimination. The chemical structure of polyphenols determines their rate and extent of absorption, as well as the chemical characteristics, such as molecular weight, lipophilicity, stereochemistry, and the presence of a group capable of hydrogen bonding (151). Representatively, low bioavailability is the major problem in the use of curcumin as a nutritional supplement. Rapid metabolism, low absorption, bio-distribution, and rapid excretion of curcumin are major challenges. Therefore, the key step that promotes its clinical transformation is solving the problem of the low bioavailability of curcumin. Solution methods include micelles, nanoparticles, liposomes, and phospholipid complexes. These methods could upregulate the bioavailability of curcumin (152). Considering the interaction between polyphenols and gut microbiota, converting polyphenols into active ingredients that can be easily absorbed and utilized through fermentation of gut microbiota can be a good way to improve their bioavailability. Moreover, developing symbiotics by mixing polyphenols with the key probiotics they regulate, is a way to accelerate their clinical application. The application of natural polyphenols in metabolic disorders, especially obesity and T2DM, is feasible and promising, with clear efficacy and potential multi-targeted mechanisms of action. They could represent an important source for the development of future drugs or dietary supplements for the management of metabolic disorders.

## Author contributions

KC, ZG, and QD were co-responsible for the collection, collation, and writing of the original manuscript. WL and LZ designed and revised the manuscript. KC, CT, HZ, TZ, WX, and ZJ were responsible for the concept development, revision, and review of the manuscript. All authors contributed to the article and approved the submitted version.
